# Lateral orbitofrontal cortex partitions mechanisms for fear regulation and alcohol consumption

**DOI:** 10.1371/journal.pone.0198043

**Published:** 2018-06-01

**Authors:** Madelyn H. Ray, Emma Hanlon, Michael A. McDannald

**Affiliations:** Department of Psychology, Boston College, Chestnut Hill, Massachusetts, United States of America; Oregon Health and Science University, UNITED STATES

## Abstract

Anxiety disorders and alcohol use disorder are highly comorbid, yet identifying neural dysfunction driving comorbidity has been challenging. Lateral orbitofrontal cortex (lOFC) dysfunction has been independently observed in each disorder. Here we tested the hypothesis that the lOFC is essential to partition mechanisms for fear regulation and alcohol consumption. Specifically, the capacity to regulate fear and the propensity to consume alcohol are unrelated when lOFC is intact, but become linked through lOFC dysfunction. Male Long Evans rats received bilateral, neurotoxic lOFC lesions or sham surgery. Fear regulation was determined by establishing discrimination to danger, uncertainty, and safety cues then shifting the shock probability of the uncertainty cue. Alcohol consumption was assessed through voluntary, intermittent access to 20% ethanol. The neurotoxic lesion approach ensured lOFC dysfunction spanned testing in fear regulation and alcohol consumption. LOFC-lesioned rats demonstrated maladaptive fear generalization during probability shifts, inverting normal prediction error assignment, and subsequently consumed more alcohol. Most novel, fear regulation and alcohol consumption were inextricably linked only in lOFC-lesioned rats: extreme fear regulation predicted excessive alcohol consumption. The results reveal the lOFC is essential to partition mechanisms for fear regulation and alcohol consumption and uncover a plausible source of neural dysfunction contributing to comorbid anxiety disorders and alcohol use disorder.

## Introduction

Anxiety disorders and alcohol use disorder are highly comorbid [1–4]. Despite this clear clinical relationship, the neural mechanisms mediating comorbid anxiety and alcohol use are only beginning to be understood. Orbitofrontal cortex (OFC) function is altered or disrupted in anxiety disorders [5, 6], as well as alcohol-use disorder [7–9]. Specifically, OFC hypoactivity is seen in people with anxiety disorders, such as PTSD [6], in addition to people with alcohol use disorder [7, 10]. This commonality may mark OFC dysfunction, via hypoactivity, as a comorbid link between anxiety disorders and alcohol-use disorder.

Consistent with dysfunction in psychiatric disorders, previous studies have demonstrated independent roles for the OFC in fear regulation [11–16] and alcohol consumption [17–19]. OFC lesions lead to generalized fear increases to shock-free contexts during contextual fear discrimination [13, 20], while OFC inhibition via DREADDs results in persistent cued fear in the face of extinction [15]. OFC lesions or DREADD inhibition increases voluntary alcohol consumption in alcohol-dependent mice [17]. Thus, the OFC normally works to reduce fear and moderate alcohol consumption.

We propose that while individually contributing to each, an essential function of the OFC, particularly the lateral OFC (lOFC), is to partition neurobehavioral mechanisms for fear regulation and alcohol consumption. A salient prediction of our hypothesis is that lOFC dysfunction should result in failure of this partition, linking the capacity to regulate fear and propensity to consume alcohol. We induced lOFC dysfunction via neurotoxic lesion in one group of rats, and left lOFC intact via sham surgery in another group. The neurotoxic lesion guaranteed OFC dysfunction across fear and alcohol testing, and provided face validity for OFC hypoactivity observed in anxiety disorders and alcohol use disorder. We established fear discrimination to cues predicting unique foot shock probabilities: danger (1.00), uncertainty (0.25), and safety (0.00). The ability to upregulate or downregulate fear was determined by increasing (p = 0.25 to p = 0.50) then decreasing (p = 0.50 to p = 0.125) the shock probability associated with the uncertainty cue in four-session blocks. Changes in fear to uncertainty, as well as generalization to danger and safety, were monitored during probability shifts. All rats were then given voluntary, intermittent access to alcohol (20% ethanol) [21], followed by voluntary access to an isocaloric control solution. Assessing fear regulation and alcohol consumption in the same subjects allowed us to directly analyze their relationship, providing an explicit test our lOFC partitioning hypothesis.

## Methods and materials

### Subjects

Subjects were forty-three male Long Evans rats weighing 275–300 g upon arrival (Charles River Laboratories; RGD Cat# 2308852, RRID:RGD_2308852). Rats were individually housed and maintained on a 12-h dark light cycle (lights off at 6:00 PM) with water *ad libitum*. Procedures adhered to the NIH Guide for the Care and Use of Laboratory Animals and were approved by the Boston College Institutional Animal Care and Use Committee.

### Surgery

Stereotaxic surgery was performed under isoflurane anesthesia (2–5%) using aseptic technique. Twenty-two rats received bilateral infusions of *N*-Methyl-D-aspartic acid (20 μg/μl in Dulbecco’s PBS) aimed at the dorsolateral subdivision of the orbitofrontal cortex (0.20 μl, +4.00 AP, ±3.60 ML, -5.10 DV; 0.25 μl, +4.00 AP, ±3.80 ML, -5.50 DV from skull) [22–24]. Infusions were delivered via 2 μl syringe (Hamilton, Neuros) controlled by a microsyringe pump (World Precision Instruments, UMP3-2). Infusion rate was ~0.11 μl/min. At the completion of each infusion, the syringe was raised 0.1 mm then left in place for five min to encourage delivery to the target site. The remaining twenty-one rats received identical surgical treatment without infusions. Rats received carprofen (5 mg/kg) for post-operative analgesia.

### Behavioral apparatus

Eight sound-attenuated enclosures each housed a behavior chamber with aluminum front and back walls, clear acrylic sides and top, and a metal grid floor. Grid floors were electrically connected to a shock generator. A single external food cup and central nose poke opening equipped with infrared photocells were present on one wall. Auditory stimuli were presented through two speakers mounted on the ceiling of each behavior chamber. Consumption testing took place in the home cage. Solutions were in 50 mL tubes with rubber stoppers and ball bearing sipper tubes [25]. Rat chow and standard water bottles were always present on the home cage during consumption testing.

### Nose poke acquisition

Following recovery from surgery, rats were food restricted to 85% of their initial free feeding weight, then fed (2–20 g/day)to increase their target body weight by 1 g/day for the remainder of testing. Rats were shaped to nose poke for pellet (BioServ F0021 –protein/fat/carbohydrate blend) delivery using a fixed ratio 1 schedule: one nose poke yielded one pellet. Shaping sessions lasted 30 min or approximately 50 nose pokes. Over the next 3, 60-min sessions, rats were placed on variable interval (VI) schedules in which nose pokes were reinforced on average every 30 s (session 1), or 60 s (sessions 2 and 3). For the remainder of testing, nose pokes were reinforced on a VI-60 schedule independent of all Pavlovian contingencies.

### Pre-exposure

In two separate sessions, each rat was pre-exposed to the three cues to be used in Pavlovian fear discrimination. Cues were auditory stimuli, 10-s in duration and consisted of repeating motifs of a broadband click, phaser, or trumpet. Stimuli can be heard or downloaded at http://mcdannaldlab.org/resources/ardbark. Previous studies have found these stimuli to be equally salient, yet highly discriminable [26–28]. The 42-min pre-exposure sessions consisted of four presentations of each cue (12 total presentations) with a mean inter-trial interval (ITI) of 3.5 min. The order of trial type presentation was randomly determined by the behavioral program and differed for each rat during each session throughout behavioral testing.

For all sessions, fear to each auditory cue was measured using a suppression ratio based on nose poke rates during the 20-s baseline period immediately preceding the 10-s cue period [28–31]: suppression ratio = (baseline nose poke rate–cue nose poke rate) / (baseline nose poke rate + cue nose poke rate). A ratio of 1 indicated complete suppression of nose poking during the cue and a high level of fear; 0, no suppression and no fear. Intermediate suppression ratios reflected intermediate fear levels.

### Initial fear discrimination

For the next sixteen sessions, each rat underwent Pavlovian fear discrimination. Each 54-min session began with a ~5-min warm-up period during which no cues or shock were presented. In discrimination, each cue was associated with a unique probability of foot shock (0.5 mA, 0.5-s): danger (1.00), uncertainty (0.25), and safety (0.00). Foot shock was administered 1-s following cue offset. A single session consisted of four danger, six uncertainty-no shock, two uncertainty-shock, and four safety trials. Mean inter-trial interval was 3.5 min.

### Increasing foot shock probability associated with uncertainty

**Fig 1:**
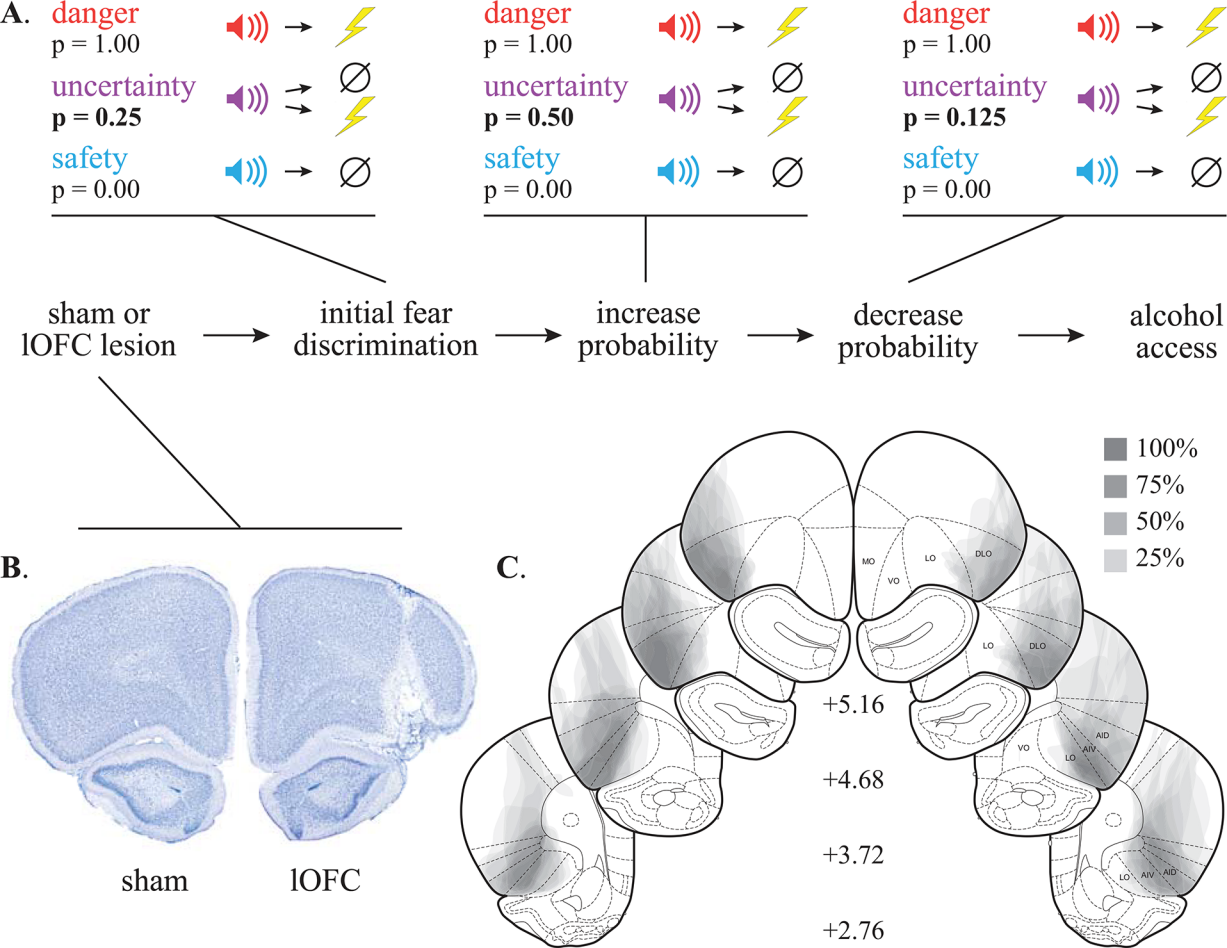
Experimental timeline and histology. **(A)** Rats received stereotaxic surgery, recovered, then underwent Pavlovian fear discrimination. The shock probability of the uncertainty cue was increased to p = 0.50 over four sessions, then decreased to p = 0.125 over four sessions. Following the completion of fear testing, rats received voluntary intermittent access to alcohol over eight sessions. **(B)** A representative control with lOFC intact (left) and lesion with OFC damage (right) is shown. **(C)** The extent of neurotoxic lOFC lesions across four coronal planes is shown, and the anterior distance from bregma (millimeters) indicated.

In order to assess rats’ ability to upregulate fear, the foot shock probability associated with uncertainty was increased from 0.25 to 0.50 starting on session 17, and continuing through session 20 (Fig 1A, middle). The total number of uncertainty trials was held constant, only now four of the eight uncertainty trials resulted in foot shock. For this and all subsequent blocks, the number and structure of the danger and safety trials were held constant, as were session length and ITI.

### Decreasing foot shock probability associated with uncertainty

In order to assess rats’ ability to downregulate fear, the foot shock probability associated with uncertainty was decreased from 0.50 to 0.125 starting on session 21, and continuing through session 24 (Fig 1A, right). Again, the total number of uncertainty trials was held constant, only now a single uncertainty trial resulted in foot shock. Finally, rats were returned to the original uncertainty foot shock probability (0.25) for sessions 25–28. All aspects of these sessions were identical to initial discrimination. Rats then underwent a single extinction session identical to pre-exposure in which each cue was presented four times but no shocks were delivered. Immediately following extinction, rats were given *ad libitum* access to home cage food and water for the remainder of the experiment.

### Voluntary alcohol consumption

Nine days following the extinction session, permitting sufficient time to overcome any food deprivation, rats received eight sessions of voluntary, intermittent, 24-h access to alcohol (20% ethanol in di H_2_0 v/v; [21]. Bottles were tested, weighed, then put on homes cages at 9:00 am on Sundays, Tuesdays, and Thursdays; and removed at 9:00 am on Mondays, Wednesdays, and Fridays. A leaky home cage water bottle was always present to ensure a constant water source, and that any alcohol consumption was voluntary. Consumption of an isocaloric, bitter, non-alcoholic solution was assessed in a ninth, 24-h session through voluntary access to quinine-adulterated DuoCal® [QuAD; 0.1% quinine (w/v), 28% DuoCal® (w/v), in di H_2_0].

### Histology

Upon the conclusion of voluntary access, rats were anesthetized with an overdose of isoflurane and perfused intracardially with 0.9% biological saline. Brains were extracted and stored in 4% (v/v) formalin and 10% (w/v) sucrose. Forty-micrometer sections were collected on a sliding microtome and Nissl-stained to verify lesion placement.

### Statistical analysis

Behavioral data were acquired using Med Associates Med-PC IV software (MED PC, RRID:SCR_012156). Raw data were processed in Matlab (MATLAB, RRID:SCR_001622) to extract nose-poke rats during two periods, baseline (20 s prior to cue onset) and cue (entire 10-s). These periods were used to calculate the suppression ratio. Suppression ratios were analyzed with repeated measures ANOVA in SPSS (RRID:SCR_002865) and Statistica (RRID:SCR_014213). Partial eta squared (η^2^) and observed power are reported for ANOVA results for indicators of effect size.

For the four-session blocks in which shock probability was altered, we calculated a fear regulation score (FRS) for each cue/subject by taking the average suppression ratio for the last two sessions of each block and subtracting the first session. This was done in order to reduce the session-by-session change in suppression ratio to a single value, critical for later linear regression and also allowing for clearer visualization of the change for each cue/group.

Consumption is reported in grams (solution consumed) / kg (body weight) per 24 hours (total time given access): g/kg/24 h. Novelty-corrected alcohol consumption was determined by subtracting QuAD consumption level from the eight-session mean of alcohol consumption. Alcohol consumption was tested for normality using the Anderson-Darling test and analyzed using the nonparametric Mann-Whitney U test, which does not assume normality.

Linear regression was performed in which FRS values from probability shift blocks (increasing and decreasing) were used as regressors to predict eight-session mean alcohol consumption. Statistical output of regression was the significance of the model (*R*^*2*^, *F*, *p* and estimate of error variance) as well as beta coefficients (β) for each regressor, with larger absolute values indicating stronger predictive relationships and β sign indicating direction of relationship. Regression models produced by sham and lOFC rats were statistically compared using the Fisher r-to-z transformation. For all analyses, *p* < 0.05 was considered significant.

## Results

Neurotoxic damage (cell loss and gliosis) was quantified for the ventrolateral, lateral, and dorsolateral subregions of the OFC. Six lOFC rats had unilateral or non-neurotoxic damage and were excluded from subsequent analyses. Sixteen lOFC rats showed damage primarily in the lateral and dorsolateral subregions of the OFC, with little damage in the ventrolateral subregion and no damage in the medial OFC. Shams showed no evidence of neurotoxic damage. Representative lOFC (Fig 1B, right), and sham (Fig 1B, left) sections are shown. Each subject’s lesion was drawn, made transparent, and stacked (Fig 1C). Darker areas indicate regions of greater overlap and more consistent damage.

**Fig 2:**
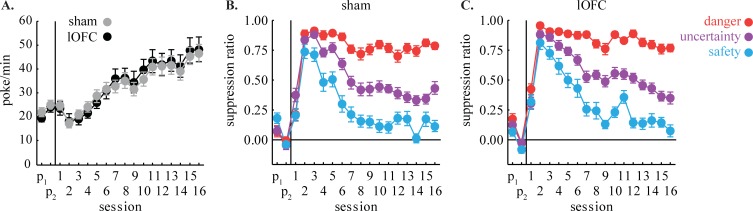
Nose poking and initial fear discrimination. **(A)** Mean ± SEM baseline nose poke rates throughout initial fear discrimination are shown for sham (gray) and lOFC rats (black). Mean ± SEM suppression ratio for danger (red), uncertainty (purple) and safety (blue) are shown for **(B)** sham and **(C)** lOFC rats for initial fear discrimination. Vertical line separates the two pre-exposure sessions (P_1_ and P_2_), from fear discrimination for each graph.

Sham and lOFC rats showed equivalent baseline nose poking rates throughout pre-exposure, discrimination, probability shift blocks, and extinction (Fig 2A). Mean ± SEM pellets earned per session: Sham, 52.25 ± 0.9 and lOFC, 51.98 ± 1.3; independent samples t test found no group difference (t_35_ = -0.57, p = 0.57). ANOVA for baseline nose poke rate with session (31) and group (sham vs. lOFC) as factors demonstrated a main effect of session [F_(30,1050)_ = 53.28, *p* = 1.69 x 10^−187^, partial η^2^ = 0.60, observed power = 1.00]. Critically, ANOVA found no main effect or interaction with group (Fs < 0.74, *p*s > 0.84). Equivalent performance ensures that differences in suppression ratios between groups cannot be attributed to differences in baseline nose poke rates.

### Initial Pavlovian discrimination

Sham and lOFC rats showed equivalent and excellent fear discrimination over the initial 16 sessions (Fig 2B and 2C). Suppression ratios were low in pre-exposure and initially increased to all cues. As discrimination proceeded, the suppression ratio for each cue diverged: high to danger, intermediate to uncertainty, and low to safety. In support, ANOVA [within factors: session (16) and cue (3); between factor: group (sham vs. lOFC)] revealed a main effect of cue [F_(2,70)_ = 154.64, *p* = 2.07 x 10^−26^, partial η^2^ = 0.82, observed power = 1.00], session [F_(15,525)_ = 52.48, *p* = 4.23 x 10^−94^, partial η^2^ = 0.60, observed power = 1.00], and a cue x session interaction [F_(30,1050)_ = 13.75, *p* = 1.05 x 10^−56^, partial η^2^ = 0.28, observed power = 1.00]. No main effect or interaction with group was observed.

### Increasing foot shock probability associated with uncertainty

**Fig 3:**
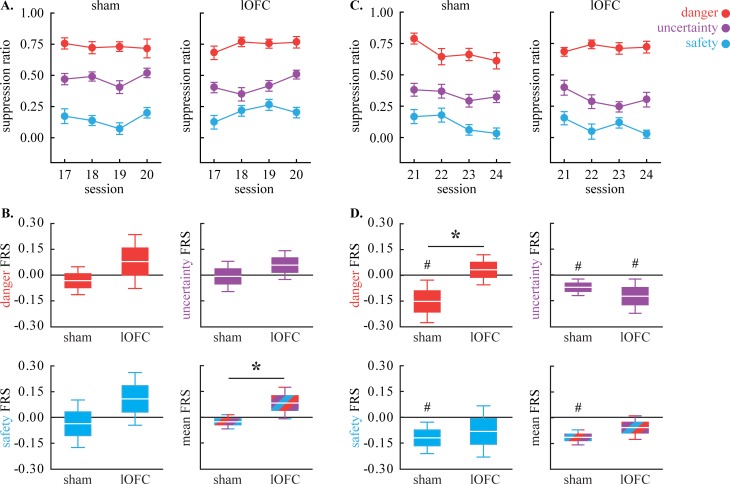
Fear regulation. **(A)** Mean ± SEM suppression ratio for danger (red), uncertainty (purple) and safety (blue) are shown for sham (left) and lOFC rats (right) for the four sessions in which the uncertainty foot shock probability was increased. **(B)** Mean ± 2 SEM (1 SEM box; 2 SEM whisker) fear regulation score (FRS) is shown for danger (top left), uncertainty (top right), safety (bottom left) and mean of all cues (bottom right); sham (left) and lOFC (right). **(C-D)** LOFC data plotted as in A-B. *independent samples, two-tailed t-test, p<0.05. ^#^single sample comparison to zero, two-tailed t-test, p<0.05.

Starting session 17, and continuing until session 20, the foot shock probability associated with uncertainty was increased from 0.25 to 0.50. Discrimination was generally maintained in both groups over the four sessions. However, shams showed little change in fear to any cue (Fig 3A, left), whereas lOFC rats tended to increase fear to all cues (Fig 3A, right). In support, ANOVA for suppression ratio [between factor: group; within factors: session (4) and cue (3)] found a main effect of cue [F_(2,70)_ = 117.91, *p* = 3.86 x 10^−23^, partial = η^2^ 0.77, observed power = 1.00] and trend toward a group x session interaction [F_(3,105)_ = 2.53, *p* = 0.062, partial η^2^ = 0.67, observed power = 0.61].

The change in fear to each cue was more apparent using the fear regulation score (FRS; Fig 3B). Shams showed FRS values around zero for uncertainty but a tendency toward negative FRS values for danger and safety. By contrast, lOFC rats showed positive FRS values to all three cues. ANOVA for FRS [between factor: group; within factor: cue (3)] found a main effect of group [F_(1,35)_ = 5.53, *p* = 0.03, partial η^2^ = 0.14, observed power 0.63]. Illustrative of the group main effect, when FRS was averaged for all three cues, a significant difference emerged with lOFC rats showing significantly higher FRS values than shams (Fig 3B, bottom right; two-tailed t-test, t_35_ = 2.34, *p* = 0.03). The lOFC is not necessary to upregulate fear to uncertainty, *per se*, but may normally *prevent* generalized increases in fear to safety and danger.

### Decreasing foot shock probability associated with uncertainty

Starting session 21, and continuing until session 24, the foot shock probability associated with uncertainty was decreased from 0.50 to 0.125. Discrimination was generally maintained. However, shams decreased fear to *all* cues (Fig 3C, left), while lOFC rats selectively reduced fear to uncertainty and safety (Fig 3C, right). ANOVA for suppression ratio [within factors: session (4) and cue (3); between factor: group (sham vs. lOFC)] found a main effect of cue [F_(2,70)_ = 81.08, *p* = 5.94 x 10^−19^, partial η^2^ 0.70, observed power 1.00], session [F_(3,105)_ = 5.95, *p* = 0.001, partial η^2^ 0.15, observed power 0.91], and a significant session x cue x group interaction [F_(6,210)_ = 2.50, *p* = 0.023, partial η^2^ 0.067, observed power 0.83].

Visualizing the FRS for each group/cue made the pattern clear (Fig 3D). FRS was consistently negative for all three cues in shams. Negative FRS values were also observed to uncertainty and safety for lOFC rats but were positive for danger. ANOVA for FRS uncovered a trend toward a cue x group interaction [F_(2,70)_ = 2.46, *p* = 0.093, partial η^2^ 0.066, observed power 0.48]. Direct comparison of FRS to danger revealed significantly lower values in sham compared to lOFC rats (Fig 3D, top left; two-tailed t-test, t_35_ = 2.28, *p* = 0.03). The lOFC is not necessary to downregulate fear to uncertainty but may normally *permit* generalized decreases in fear to danger.

### Alcohol consumption

**Fig 4:**
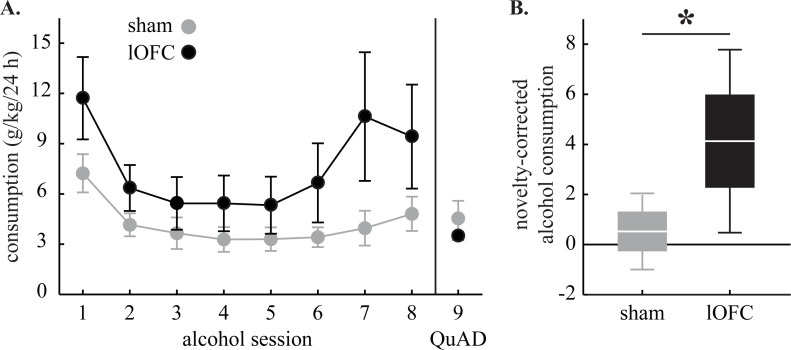
Voluntary alcohol consumption. **(A)** Mean ± SEM consumption (g/kg/24h) for the eight alcohol sessions and single QuAD session is shown for sham (gray) and lOFC rats (black). **(B)** Mean ± 2 SEM (1 SEM box; 2 SEM whisker) novelty-corrected alcohol consumption is shown for sham (gray) and lOFC rats (black). *Mann-Whitney U, p<0.05.

Alcohol consumption was non-normally distributed in sham [A2 = 2.15, *p* = 5.00 x 10^−4^] and lOFC rats [A2 = 1.46, *p* = 5.29 x 10^−4^]. While there was variability, consumption levels were higher in lOFC rats in each of the eight alcohol sessions (Fig 4A). Increased consumption by lOFC rats was restricted to alcohol, as low and similar levels of QuAD consumption were observed in both groups. Mann-Whitney U for 8-session mean alcohol consumption revealed a trend toward greater consumption by lOFC rats [U = 112, *p =* 0.089] but found significance for novelty-corrected alcohol consumption [U = 91, *p* = 0.018] (Fig 4B).

### Predictive relationship between fear regulation and alcohol consumption

If the lOFC partitions fear regulation from alcohol consumption, then we should be able to use fear regulation to uncertainty–the only cue for which shock probability was altered–to predict alcohol consumption only in lOFC rats. The uncertainty FRSs from increasing shock probability (data from Fig 3B, top right) and decreasing shock probability (data from Fig 3D, top right) were used as regressors to predict novelty-corrected alcohol consumption (data from Fig 4B). Novelty-corrected alcohol consumption was z-score transformed (mean = 0, standard deviation = 1) in order to relate the change in FRS to standard deviation change in consumption.

**Fig 5:**
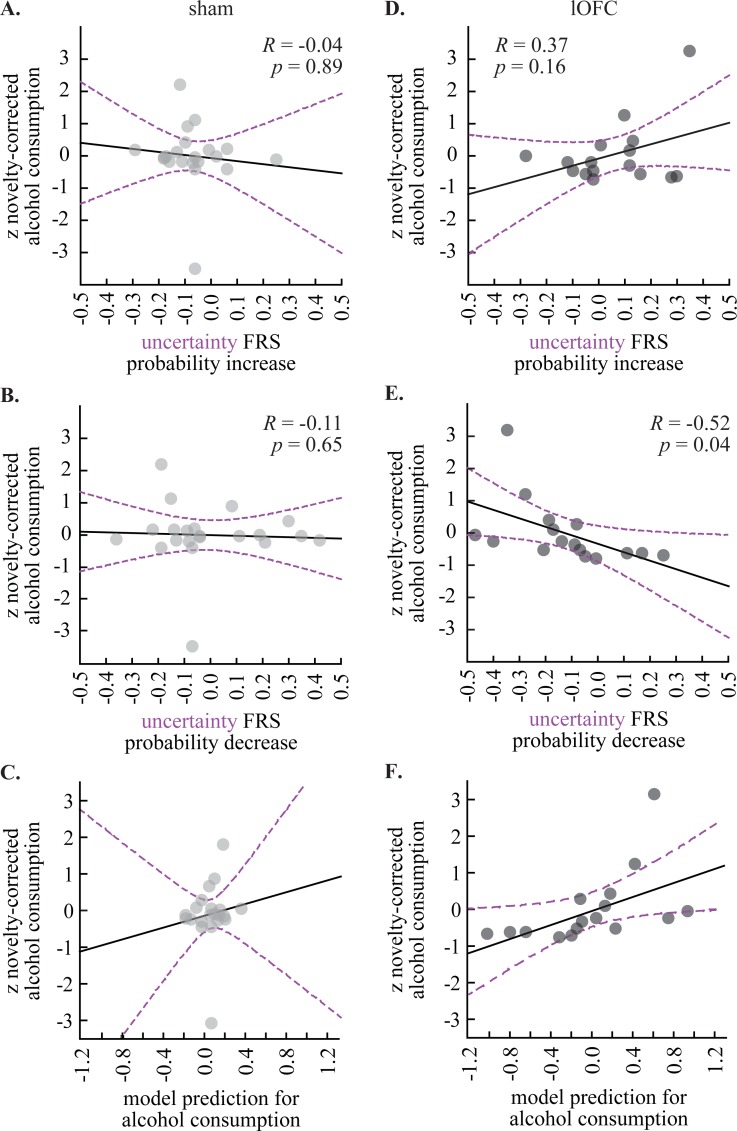
Relationship between fear regulation and voluntary alcohol consumption. Scatterplots show the **(A)** correlation between uncertainty FRS for shock probability decrease (x-axis) and the z-score for novelty-corrected alcohol consumption (y-axis) and **(B)** correlation between uncertainty FRS for shock probability increase (x-axis) and the z-score for novelty-corrected alcohol consumption (y-axis) for shams. Correlation coefficient (*R*) and significance (*p*) are indicated for each plot. **(C)** Regression model prediction for alcohol consumption (data from A and B) is compared to observed alcohol consumption for shams. Trendline (solid black) and 95% confidence interval (dashed purple) are plotted for each scatterplot. **(D-F)** LOFC data plotted as in A-C.

We were unable to use uncertainty fear regulation to predict alcohol consumption in shams (regression model: *R*^*2*^ *=* 0.02, *F* = 0.15, *p* = 0.87, error = 1.09). Indeed, there was near zero correlation between upshift FRS and novelty-corrected alcohol consumption (Fig 5A) and near zero correlation between downshift FRS and novelty-corrected alcohol consumption (Fig 5B). The resulting regression model had considerable error and did not fit the observed pattern of alcohol consumption (Fig 5C). Similar failures were observed when using danger FRSs (regression model: *R*^*2*^ *=* 0.05, *F* = 0.45, *p* = 0.64, error = 1.06), safety FRSs (regression model: *R*^*2*^ *=* 0.04, *F* = 0.38, *p* = 0.69, error = 1.06), or a single model containing all six FRSs (regression model: *R*^*2*^ *=* 0.16, *F* = 0.46, *p* = 0.83, error = 1.19). Sham regression also failed if uncorrected alcohol consumption was predicted (regression model: *R*^*2*^ *=* 0.03, *F* = 0.27, *p* = 0.77, error = 1.08). A power analysis revealed that 490 sham subjects would be necessary to observe a predictive relationship between uncertainty fear regulation and alcohol consumption (p < 0.05, power ≥ 0.80). Simple correlations revealed zero relationship between novelty-corrected alcohol consumption the upshift FRS (*R = -*0.04, *p* = 0.89; Fig 5A) and downshift FRS (*R =* -0.11, *p* = 0.65; Fig 5B).

By contrast, uncertainty fear regulation predicted alcohol consumption in lOFC rats (regression model: *R*^*2*^ *=* 0.63, *F* = 10.84, *p* = 0.0017, error = 0.43, observed power 0.97). The beta coefficient for FRS during upshift (β = 3.78) and FRS during downshift (β = -3.75) were equivalently large, but in opposing directions. The resulting regression model had less error, and better fit the observed pattern of alcohol consumption (Fig 5F). The predictive relationship between FRS and alcohol consumption was restricted to uncertainty, as individual analyses for danger (regression model: *R*^*2*^ *=* 0.10, *F* = 0.73, *p* = 0.50, error = 1.04) and safety (regression model: *R*^*2*^ *=* 0.20, *F* = 3.30, *p* = 0.23, error = 0.92) did not reveal significant models. A virtually identical result was found for lOFC rats when using uncertainty FRSs to predict uncorrected, 8-session mean alcohol consumption (regression model: *R*^*2*^ *=* 0.61, *F* = 10.12, *p* = 0.002, error = 0.45). The Fisher r-to-z transformation revealed a significant difference between r values in the lOFC and sham groups for novelty-corrected alcohol consumption (Z = 2.60, *p* = 0.0099) and uncorrected alcohol consumption (Z = 2.40, *p* = 0.016). The lOFC model produced a significantly stronger correlation coefficient and, therefore, greater fit than the sham model. When simple correlations were examined, the upshift FRS was positively, but not significantly, correlated with novelty-corrected consumption (*R =* 0.37, *p* = 0.16; Fig 5D), while the downshift FRS was significantly, negatively correlated with novelty-corrected consumption (*R =* -0.52, *p* = 0.04; Fig 5E).

## Discussion

### LOFC contribution to fear regulation

The lOFC was not required for mastery of initial fear discrimination. Somewhat surprisingly, shams showed little change in fear to uncertainty–or any cue–when shock probability was increased. Extensive training of the initial discrimination may have meant that more than four sessions would be required to increase fear to uncertainty. By contrast, lOFC rats increased fear to all three cues. Only in lOFC-damaged rats, when the uncertainty cue worsened, all cues worsened. Shams readily decreased fear to uncertainty when the foot shock probability was decreased, generalizing this decrease to safety and danger. LOFC rats decreased fear to uncertainty, generalized to safety, but failed to generalize to danger. Only in lOFC-intact rats, when the uncertainty cue improved, all cues improved. This overall pattern is consistent with findings from the reward literature, in which OFC lesions typically leave the acquisition of discrimination intact, but alter performance during reversal and the ability to track changes in cue-reward contingencies [32–39].

Of course, we cannot rule out the possibility that lOFC lesions altered cue encoding during initial fear discrimination, despite leaving behavior intact, and that probability shifts only revealed this deficit. For example, lOFC lesions could have increased sensitivity to uncertainty, making these rats more responsive to probability changes in either direction. Yet, fear to uncertainty–the only cue experimentally altered–never significantly differed between sham and lOFC rats at any point. Only differences to safety and danger were observed, and these only occurred during probability shifts. LOFC lesions could have made the cues more difficult to separate, but this should have resulted in uniform changes across all cues during upshift and downshift, instead of the asymmetrical generalization pattern we observed. The most parsimonious account of the overall pattern of fear is that lOFC lesions did not impair encoding or separation of cues during initial discrimination, but altered fear generalization during probability shifts.

The role of the lOFC in fear regulation may be conceptualized by the reward construct of ‘credit assignment’ [39, 40]. Credit assignment reflects the need to attribute discrepancies between expected and received rewards (prediction errors) to specific causes (e.g. cues or choices) [41]. If one makes a series of choices (A, B, and C), and only choice C produces more reward than expected, credit needs to be selectively assigned to C. Rhesus macaques with OFC intact correctly assign credit (to choice C), whereas macaques with OFC damage randomly assign credit (to choices A, B and C) [39]. Viewed through the lens of credit assignment, increasing shock probability induces a positive error, and in normal rats, positive errors are selectively assigned to uncertainty. Decreasing shock probability induces a negative error that is globally assigned. LOFC damage inverts this assignment; positive errors are now globally assigned, while negative errors are more selectively assigned. The net result of the lOFC lesion-induced inversion is to more globally enhance or maintain fear when a variable predictor of threat worsens *or* improves.

A role for the lOFC to regulate prediction error assignment is accordant with previous findings. Rats receiving OFC inactivation with muscimol following discriminative cue conditioning show excessive fear to safety in a recall test [42]. Rats with OFC lesions fail to acquire discriminative fear to a context paired with shock versus a shock-free context, showing excessive fear to shock-free context [13, 20]. In terms of prediction error assignment, normal rats selectively assign positive prediction error (surprising shock receipt) to the shock context, while OFC-lesioned rats inappropriately assign positive error to the shock-free context, as well as the shock context. Further, it has been shown that OFC lesions [20] or OFC DREADD inactivation [15] result in sustained cued fear in the face of extinction. This is consistent with inappropriate assignment of negative prediction error when shock is surprisingly omitted in extinction. Use of inhibitory DREADDs [15] specifically during probability shift sessions would have permitted direct demonstration of the lOFC contribution to prediction error assignment. However, this temporally-focused approach would not have permitted disruption of lOFC function spanning fear regulation and alcohol consumption, a requirement for testing the partitioning hypothesis. Our hypothesized role for the OFC in prediction error assignment unites function in fear and reward settings and is consistent with findings from the fear conditioning literature.

### LOFC contribution to alcohol consumption

LOFC lesions increased alcohol consumption over that of an isocaloric control solution. This finding is in general accordance with a recent study showing that lOFC DREADD inhibition or lesions increased alcohol consumption in alcohol-dependent mice [17]. Our finding that the lOFC regulates alcohol consumption is line with work demonstrating that alcohol drinking induces OFC opioid release in people, and alterations in OFC binding correlate with excessive alcohol consumption [19]. One caveat is that we did not measure drinking from the water bottle. The water bottle was designed to be leaky to ensure any and all alcohol consumption was entirely voluntary. Thus, the present data cannot speak to a preference for alcohol over water, nor can we entirely rule out lOFC lesions affecting overall fluid consumption. The pattern of consumption in the current study, initially high levels of consumption followed by a decrease across sessions, differs from other reported patterns of consumption where intake escalates over time [21, 43]. However, differences in observed drinking patterns are not uncommon and can be due to a variety of methodological differences such as age, bottle type, housing conditions, and the nature of previous alcohol experience [25, 44]. The pattern we observed is highly consistent with previous reports from our laboratory irrespective of [27, 45, 46] and following fear discrimination [47]. OF course, we cannot rule out that prior fear conditioning influenced alcohol consumption. The current results support a role for the lOFC in moderating and reducing voluntary alcohol consumption. Behavioral procedures assessing alcohol learning [48], as well as motivational properties of alcohol [46] and alcohol-associates cues [49], are likely to uncover an even greater role for the lOFC.

### LOFC partitions mechanisms for fear regulation and alcohol consumption

Fear regulation and alcohol consumption became inextricably linked when the lOFC was damaged. Extreme increases in fear to uncertainty following upshift, and extreme decreases in fear to uncertainty following downshift, predicted excessive alcohol consumption. This is in contrast to the clear partitioning exhibited by lOFC-intact shams. While admittedly idiosyncratic, the lOFC as partitioner is broadly consistent with a proposal for the OFC as a ‘cognitive map’ [50, 51]. The cognitive map view stems from findings that, within a structured reward task, the OFC organizes and separates task-relevant components. This includes observable events [52] and hidden states, which must be inferred from the task structure [50, 53]. This map is sufficiently detailed such that the OFC informs the current ‘location’ within the structure [50]. Partitioning is inherent to distinguishing task-relevant from task-irrelevant information and discerning location within a larger cognitive map.

Our proposal can be seen as the application of the cognitive map hypothesis to a more macro level. The partitioning hypothesis, and our data, suggest that a primary result of lOFC damage is to alter information processing pertaining to fear and alcohol in a larger neural network. LOFC projects to a variety of brain regions critical to processes of fear *and* alcohol, including prelimibic cortex [54–56], basolateral amygdala [45, 57, 58], central amygdala [58–60], and nucleus accumbens [61–63]. Just as the lOFC organizes and separates information within a single reward task, the lOFC may organize and separate information across distinct motivational mechanisms (fear versus alcohol) in single neurons, or neural ensembles, in a wider neural network. A primary result of reduced OFC function would be common processing of fear and alcohol information in a wider neural network.

### LOFC and comorbid anxiety disorders/alcohol use disorder

Our results provide mechanisms by which OFC dysfunction (hypoactivity) could individually contribute to each disorder. For anxiety disorders and stress disorders [64], OFC hypoactivity would encourage generalization of aversive prediction errors in a way that increases fear, or maintains fear, when a variable threat predictor became more *or* less predictive. For alcohol use disorder, OFC hypoactivity would promote alcohol consumption and/or alcohol seeking. Most novel, OFC hypoactivity may be a key mechanism by which neurobehavioral circuits for fear and alcohol, which are normally partitioned, become linked. OFC dysfunction initially triggered by disordered anxiety could drive excessive alcohol consumption, and/or OFC dysfunction initially triggered by disordered drinking could drive extreme fear regulation. A testable prediction of this hypothesis is that that greater OFC hypoactivity (during a fear task, alcohol task, or at baseline) would be found in individuals with comorbid anxiety disorder/alcohol use disorder, compared to those with only one disorder or the other. Even more, fear-related and alcohol-related cues/processes should produce more similar patterns of network activity in comorbid individuals than health controls or individuals with only one disorder.

## Conclusion

Here we have revealed the lOFC is essential to partitioning of fear regulation and alcohol consumption. These results provide a plausible source of neural dysfunction that directly contributes to anxiety disorder and alcohol use disorder independently and most notably, their comorbid relationship. Future studies examining fear and alcohol information processing in amygdalar, striatal, and prefrontal neural ensembles [65] with lOFC intact or impaired, will provide valuable insight into normal lOFC partitioning. Such studies are likely to accelerate understanding of the neural dysfunction driving comorbidity of anxiety and alcohol use disorder, hastening the development of therapies to restore function.
